# COVID-19 Vaccination in Migrants and Refugees: Lessons Learnt and Good Practices

**DOI:** 10.3390/vaccines10111965

**Published:** 2022-11-19

**Authors:** Palmira Immordino, Davide Graci, Alessandra Casuccio, Vincenzo Restivo, Walter Mazzucco

**Affiliations:** Department of Health Promotion, Mother and Child Care, Internal Medicine and Medical Specialties, 90127 Palermo, Italy

**Keywords:** immunization, vaccines, health policies, migrants, refugees, vulnerable groups, COVID-19, global health

## Abstract

The COVID-19 pandemic has exacerbated inequalities between low- and high-income countries. Within the latter, a greater impact is seen in the poorest and most vulnerable people, including refugees, asylum seekers, and migrants. They all may experience poor access to quality healthcare or have suboptimal health-seeking behavior, distrust of governments, or fear of detention and deportation if seeking healthcare. Some refugees and migrants may face multiple barriers to vaccination and access to health systems that are relevant to the administration of COVID-19 vaccines, despite the growing inclusion of these populations in public health policies. Several good practices have emerged to ensure the inclusion of these populations in vaccination and healthcare for COVID-19 globally. However, inequalities persist between high-income and low-/middle-income populations. The inequalities in COVID-19 vaccination reflect the already existing ones in common health services worldwide. Further efforts are necessary to reduce such disparities, to protect the vulnerable, and, by extension, the general population. The initiatives organized, both at global and local levels, to support vaccination campaigns represent a notable example of how complex multilevel structures, such as health systems, as well as limited resource health services, can successfully face, even during a health emergency, the challenges related to global health issues.

## 1. International Policy Framework on COVID-19 Vaccination in Migrants and Refugees

The COVID-19 pandemic has exacerbated inequalities between low-income countries (LICs) and high-income countries (HICs) as defined by the World Bank, (Country classification by income (https://datahelpdesk.worldbank.org/knowledgebase/articles/906519, accessed on 23 September 2022)). Within the latter, a greater impact is seen in the poorest and most vulnerable people, including refugees, asylum seekers, and migrants (In this article we refer to migrants, refugees, and asylum seekers as they are defined by the relevant UN agencies. Source: International migration law: glossary on migration. Geneva: International Organization for Migration; 2019 (Report No. 34; https://publications.iom.int/system/files/pdf/iml_34_glossary.pdf, accessed on 23 September 2022) and Convention and protocol relating to the status of refugees. Geneva: Office of the United Nations High Commissioner for Refugees (UNHCR); 1951 (United Nations General Assembly Art. 1(A)(2); https://www.unhcr.org/3b66c2aa10.pdf, accessed on 23 September 2022)) [1].

Social, political, and economic exclusion can cause and accentuate the phenomena of poverty and exploitation, with a higher risk of infection with SARS-CoV-2 and other vaccine-preventable diseases [2]. Indeed, refugees and migrants are often forced to live in neighborhoods with high housing densities or to work in inadequately protective conditions (such as seasonal migrants in informal camps, overcrowded dormitories, receptions, detention centers, etc.) [3,4], with limited capacity for physical distancing and self-isolation [5,6]. Regular migrants (those who entered a country legally and remain there according to the admission criteria in place) working in essential sectors with greater exposure to the virus are, as a result, more vulnerable to infection than the general population as well [7]. They all may experience poor access to quality healthcare or have suboptimal health-seeking behavior, distrust of governments, or fear of detention and deportation if seeking healthcare. Studies conducted in different countries of the Organization for Economic Co-operation and Development (OECD) have found a risk of infection in these groups that is at least double compared to that in the general population [7,8].

Both the COVID-19 Strategic Plan for Preparedness and Response (WHO, 2021) and the global humanitarian response plan to the pandemic (United Nations, 2020) emphasize the needs of the most vulnerable groups of the population [9,10]. In addition, the Security Council of the United Nations (UN, 2021) has called for an international cooperation agreement to facilitate equal access to COVID-19 vaccines, even for those countries that are in conflict or experiencing complex humanitarian emergencies [11]. Regarding prioritizing the use of COVID-19 vaccines in the context of limited supply, the WHO Strategic Advisory Group of Experts (SAGE) have recommended considering the risks and needs of vulnerable groups, including refugees, asylum seekers and migrants, includinglow-income migrant workers, migrants in irregular situations, and people in situations of conflict or affected by humanitarian emergencies who—due to underlying social, geographical, or biomedical factors—are at higher risk of serious illness and death due to COVID-19 [12]. More recently, the WHO released a new operational guide to promote COVID-19 vaccination uptake and tackle vaccine hesitancy among refugee and migrant populations through a range of strategies, actionable recommendations, and good practices for understanding and addressing barriers [13]. This guide states that vaccine prioritization within countries should include refugees and migrants, while calling for affordable non-discriminatory access to vaccines for all populations.

As of 21 September 2022, it was estimated that 72.5% of the population in HICs had been vaccinated for COVID-19 with at least one dose, while in LICs only 23.9% of the resident population had received at least one shot [14]. This lack of fairness in vaccinations may, in part, explain the highest numbers of excess deaths due to COVID-19 being estimated in South Asia, North Africa, and the Middle East [15]. Furthermore, it has been estimated that LICs would need to increase their healthcare spending by an average of 56.6% to cover the vaccination costs of 70% of their population [14].

When the vaccine roll-out started, the International Organization for Migration (IOM) reported that only regular migrants were included in vaccination campaigns, while in many countries irregular migrants were not [16]. Recent updates from the UNHCR report that 162 countries have included refugees in their national COVID-19 vaccine plans; however, information about the inclusion of irregular migrants in the different local vaccination programs is scanty [17].

Some refugees and migrants (particularly migrants in irregular situations) may face multiple barriers to vaccination and access to health systems relevant to the administration of COVID-19 vaccines, despite the growing inclusion of these populations in public health policies. These barriers may include limited vaccine supply; low confidence in the benefits and safety of the vaccine; social influence and norms; lack of information on how to obtain vaccines; language barriers; complex registration processes and limited access to the web; and fear of arrest, detention, or deportation [18,19]. Moreover, operational and administrative barriers (such as identification documents and residence permit) can hinder migrants’ access to vaccines as well [20]. Stigma, discrimination, exclusion, and lack of access to health information and quality healthcare all represent additional barriers [21], and these factors can exacerbate any distrust in government while creating alienation from public health services. Lastly, the lower rates of vaccination coverage in refugees and migrants as compared to the general population may be also explained by skeptical attitudes towards vaccination [22,23], making them susceptible to vaccine hesitation [24], which can be addressed with clear, accessible, and tailored information campaigns [25].

The WHO Apart Together study found that, among thosesurveyed, not all migrants would seek medical care if they suspected COVID-19 infection; this was for a variety of reasons, including lack of financial means, fear of deportation, lack of availability of healthcare providers, or lack of entitlement to healthcare. Better program design is therefore considered necessary to meet their specific needs [26]. A survey conducted in June 2021 by 52 national Red Cross and Red Crescent societies found that 90% reported the lack of information or awareness on where and how to access COVID-19 vaccines as a key barrier for migrants, while 67% identified language barriers.

Another study investigated, through a qualitative methodology consisting of in-depth semi-structured interviews, the perspectives of migrants residing in the UK for less than 10 years, including refugees, asylum seekers, undocumented migrants (visa overstayers, rejected asylum seekers, and others without documentation), and individuals with limited residence permits [25]. Participants said that they had historically relied on charities for help with healthcare and general practitioner (GP) registration, and that the disruption or digitization of many of these services during lockdowns and isolation has heavily impacted them by affecting their ability to register with a family doctor. Migrants who arrived in the UK less than two years before reported difficulties in GP registering using the National Health System (NHS) digital procedure that was put in place during the pandemic. Difficulty understanding the NHS system on arrival and poor treatment by staff during the registration processes have been reported by asylum seekers and refugees as factors affecting their trust in health services. Furthermore, many respondents were hesitant to be vaccinated due to concerns about potential side effects and insufficient experimental tests during clinical trials to ensure its safety. Some even refused the vaccine for religious reasons.

## 2. Acting in the Global and Local Contexts

The COVAX program, one of the three pillars of the Access to COVID-19 Tools (ACT) Accelerator collaboration, was jointly launched in response to the pandemic in April 2020 by the WHO, the European Commission, and France. It specifically aims at providing equal and universal access to vaccines by bringing together governments, global health organizations, manufacturers, scientists, the private sector, civil society organizations, and philanthropic groups to provide innovative and equitable access to COVID-19 diagnostics, treatments, and vaccines [27]. As of July 2022, COVAX has mainly been working on delivering sufficient vaccines to 34 low–middle-income countries (LMICs) eligible for free vaccine supplies [28]; nevertheless, only a few countries around the world have active vaccination programs that specifically include migrants and refugees.

In late August 2021, the WHO published the guide “COVID-19 immunization in refugees and migrants: principles and key considerations”. This document is based on a comprehensive review of the National Distribution and Vaccination Plans submitted to the COVAX initiative, and concerns the inclusion of refugees and migrants, the experience derived from their implementation, and the introduction of COVID-19 vaccines all over the world. The document also provides information on the main challenges and barriers to accessing vaccination services, key considerations for addressing them, and presents examples of good practice [29].

In all six WHO Regions, several good practices have emerged to ensure the inclusion of refugees and migrants in vaccination and healthcare for COVID-19.

In the WHO Region of the Americas, most countries have included refugees and other displaced persons in their vaccination implementation program. A review from July 2021 which analyzed, summarized, and compared the policies regarding migrants and refugees in the Latin American countries’ vaccination plans, showed some peculiar differences [30]. In Argentina, according to the Plan Estratégico de Vacunación contra COVID-19 (Strategic Vaccination Plan against COVID-19), once high-priority categories had been vaccinated (including health workers, armed forces, teachers, older people, and people with comorbidities), subsequent prioritization would be based on vulnerability conditions, including migratory status [31]. In Ecuador, after migrants were included in the third phase of the campaign (between July and August 2021), individuals with irregular migratory status can register with the help of the United Nations High Commissioner for Refugees to get vaccinated [32]. In Brazil, even though access to health coverage is provided regardless of migratory status, irregular migrants did not qualify as a priority category and did not receive proper instructions on how to access vaccines [33]. In Chile, despite the Ministry of Health granting vaccination for everyone regardless of migratory status, the vaccination plan explicitly excluded people with a transitory visa, and the vaccination guidelines did not contain any information on the inclusion of migrants, causing contradiction and confusion regarding their eligibility for COVID-19 vaccines [34,35]. In Colombia, vaccination is granted for irregular migrants who have registered with the Estatuto Temporal de Protección para Migrantes, and a 10-year temporary protection status has been provided to Venezuelan migrants, which would allow them to register for vaccination [36]. In Peru, irregular migrants were not mentioned in the vaccination plans, leaving healthcare providers the ability to determine whether they could get vaccinated [37]. Meanwhile, in the United States of America, no identification is required to receive the vaccine [38].

Most countries in the WHO African and Eastern Mediterranean region have started to include refugees in their vaccination programs. Senegal has allowed refugees to register in designated centers near their communities. Another notable example comes from Kenya, where the third phase of the vaccination campaign included vulnerable categories, such as prisoners, refugees, and the elderly. At the same time, information was provided and healthcare was strengthened around treatment and testing within refugees’ camps [39]. Likewise, in Sudan, the government has stated that asylum seekers and displaced people at high risk should have access to vaccines [40]. Similarly, the Rwandan government has prioritized asylum seekers and refugees alongside health workers, the elderly, teachers, prisoners, and people with underlying chronic diseases in its vaccination plan [41]. Furthermore, to estimate stocks of vaccinations needed, the government has conducted rapid national screening to update data about population demographics and non-communicable diseases through a digital record-keeping program [42]. In Uganda, the Minister of Health has promoted equal access for refugees, migrants, and citizens; however, ignorance of the policy by the health workers, misinformation, or language barriers have negatively affected the access of refugees to the vaccines [43]. In contrast, it appears that migrants and refugees in Southern Africa have been left out of the vaccination plans until at least the summer of 2021 [39]. In Morocco, the government has allowed irregular migrants to regularize their position to access COVID-19 vaccination [44]. In Libya, the government has been willing to cover the costs of rolling out a COVID-19 vaccination scheme for over 570,000 refugees, but not the cost of the vaccines themselves [39].

A report about the Eastern Mediterranean Region highlights how Kuwait, Saudi Arabia, and United Arab Emirates, where healthcare is granted to regular migrants (either by the government or by their employers), have allowed irregular migrants to regularize their position without paying a fine in the wake of the COVID-19 pandemic. In Egypt, starting in the spring of 2021, irregular migrants can register with the UNHCR and access COVID-19 vaccinations via the online registration form provided by the Ministry of Health and Population [44]. By applying the concept of inclusivity and equity to access healthcare services, the Jordanian government has made an important step towards universal health coverage by using the same priority criteria (health workers, elderly people, and people with underlying health conditions) for everyone, regardless of nationality and/or state of residence, since the beginning of the pandemic. In Jordan, about 85.0% of the Syrian refugee population in 2018 were highly or severely vulnerable [45] because they lived below the poverty line [46]. As of the end of May 2021, 30% of refugees eligible to receive COVID-19 had received at least the first dose [47]. 

In the regions of Southeast Asia and the Western Pacific, countries such as Australia, Bangladesh, Malaysia, the Maldives, and Thailand have included refugees and migrants in their National Deployments Vaccination Plans (NDVPs) [29].

Several HICs in the WHO European Region have announced that they will vaccinate all the people in their territory. Some countries, such as the Republic of Moldova and Serbia, have delivered vaccines to asylum centers. Some others (Italy, France, the UK, Belgium, the Netherlands, Finland) have vaccination programs that include undocumented people; however, it remains unclear how these people can access vaccinations. To date, official data needed to quantify the number of vaccinated refugees and migrants are not available. In Greece, while refugees and asylum seekers can access vaccines, there is no clear provision for undocumented people [48]. In France, a vaccination campaign aimed at the homeless and migrants highlighted the difficulties in following up for the second dose for those people who changed shelters after the first dose [49].

Interestingly, to safeguard public health and to ensure the maximum extension of the COVID-19 vaccination campaign in favor of migrants arriving in Italy, local health authorities were called in late August 2021 to intensify measures to vaccinate people living in poverty or not registered in the National Health System (nor possessing European Health Insurance Card), and to allow access to vaccination by using an alternative code, such as the ones for Temporarily Present Foreigners (STP) or Not Registered Europeans (ENI) [50]. On the island of Sicily, which represents one of the main entry gates to Europe for migrants arriving from Africa through the Mediterranean Sea [51,52], several vaccination strategies have been implemented to vaccinate migrants and refugees hosted at migration camps and quarantine vessels [53,54,55]. Experiences of multiculturally sensitive approaches were adopted to organize and implement vaccination campaigns, including separated areas and paths for women assisted by female health personnel only [56].

## 3. Future Perspectives: GLOCAL Approach

At a global level, inequalities persist between HICs and LMICs; the former have received 15 times more doses per capita compared to the Sub-Saharan countries and LICs, and have surpassed their actual need. To counteract these criticisms, wealthy countries have pledged to donate part of their excess supplies to LMICs through COVAX, but approximately one-quarter of them have been provided to COVAX (356 million over 1.3 billion doses) [57]. In this framework, a major gap persists in terms of data collection and information sharing on vaccination uptake and immunization coverages. It is essential to invest in monitoring systems to fill this information gap in vulnerable groups, particularly in undocumented migrants. Data information systems and data sharing should be improved to document processes and outcomes related to vaccine uptake among vulnerable groups.

Despite the above-mentioned efforts documented in different countries and areas, many socially vulnerable categories remain excluded from access to health services, including vaccination, worldwide. 

In some vaccination campaigns, major challenges appear related to lack of proper information, mistrust, and/or language barriers, reducing the access of vulnerable individuals to the vaccination. For this reason, the support of local associations, cultural mediators and interpreters, and community and religious leaders is crucial to reach as many people as possible. Indeed, the use of religious leaders (priests, imams, and leaders of other faith communities) may raise awareness about COVID-19 vaccination campaigns, helping to communicate, in an effective way, the logistical information to implement the campaign. These leaderships must be taken into consideration to engage with migrant communities living in the different metropolitan areas to overcome existing cultural and religious barriers to accessing vaccination.

Moreover, a more feasible approach should involve national and international non-governmental organizations (NGOs) working closely with local health authorities and academia. These groups have been at the forefront at all levels of the COVID-19 response, from risk communication and health prevention initiatives, to procurement of vaccines and outreach activities to migrants and refugees.

In conclusion, the inequalities in COVID-19 vaccination reflect the already existing ones in access to health services worldwide, from prevention, to diagnosis and treatment. In Italy, in the early phase of the pandemic, evidence have shown that the hospitalization rates were higher in undocumented foreigners, and that they were most likely to present a more severe clinical outcome compared to Italian nationals [58]. 

If non-nationals are hindered in accessing healthcare services in a timely manner, testing, tracking, and tracing policies can be delayed with a possible negative impact on individual outcome and on disease prevention and control at population level [59]. 

It appears rhetorical to highlight how further efforts are necessary to reduce such disparities, to protect the vulnerable, and, by extension, the general population. The initiatives organized, both at global and local levels, to support vaccination campaigns represent a notable example of how complex multilevel structures, such as health systems, as well as limited resource health services, can successfully face, even during a health emergency, the challenges related to global health issues. Therefore, a GLOCAL approach should be promoted to disseminate the above-mentioned successful experiences in response to COVID-19, and also to other vaccine-preventable diseases, while suggesting the development of a compendium of good practices highlighting both the organizational aspects to be promoted and the results achieved.

## Data Availability

Not applicable.
